# Enhanced Mechanical and Water Absorption Properties of Rice Husk-Derived Nano-SiO_2_ Reinforced PHBV Composites

**DOI:** 10.3390/polym10091022

**Published:** 2018-09-13

**Authors:** Li Wang, Yong Guo, Yuxia Chen, Tong Chen, Shiliu Zhu, Tingting Zhang, Shengquan Liu

**Affiliations:** College of Forest and Garden, Anhui Agricultural University, Hefei 230036, China; 18356092360@163.com (L.W.); sheherose@163.com (Y.C.); 18356092289@163.com (T.C.); zhuslwood@163.com (S.Z.); 18715068105@163.com (T.Z.)

**Keywords:** rice-husk, SiO_2_, RHNS, PHBV, mechanical properties, water absorption, thermal stability

## Abstract

Poly(3-hydroxybutyrate–*co*–3-hydroxyvalerate) (PHBV) is a biodegradable polymer and has several potential applications. Herein, we have used a rich biomass resource, rice husk, to obtain rice husk nano-SiO_2_ (RHNS) and prepared RHNS/PHBV composites by using hot-press molding. The results showed that the amorphous nature of spherical nano-SiO_2_ particles with an average diameter of 40–80 nm was obtained. The tensile strength and flexural strength of the RHNS/PHBV-3 composite reached up to 23.515 and 75.669 MPa, respectively, corresponding to an increase of 33.65% and 15.54% as compared to pure PHBV. The enhanced mechanical properties of the RHNS/PHBV composite can be attributed to the uniform dispersion and strong interfacial bonding of RHNS with the PHBV matrix. In addition, the water absorption rate of the RHNS/PHBV composite increased from 0.26% to 0.35% and the water swelling ratio followed the given order in different directions: thickness > width > length. Furthermore, the initial degradation temperature and residual rate of combustion at 700 °C of the composites increased with higher content of RHNS, which represents the enhanced thermal stability of RHNS/PHBV composites. In summary, RHNS served as an excellent reinforcement and RHNS/PHBV composites have shown promising properties for various potential applications.

## 1. Introduction

Annually, more than 600 million tons of rice is produced worldwide, and the weight of rice husk accounts for 20% of the total weight [1]. Rice husk, as a by-product of rice processing, has a large yield and is easy to collect. As an agricultural waste, the rice husk is cheap, rich in lignin, cellulose and silica, and offers advantages of low density and high degradability [2]. Hence, rice husk is considered for a wide range of applications. At present, the rice husk is being investigated for the preparation of combustible gases [3], adsorbents [4] and catalysts [5], activated carbon [6], chemical products (i.e., xylose [7] and furfural [8]), and various silicon products [9]. Moreover, rice husk has shown potential in the field of composite reinforcements [10,11]. However, a large amount of rice husk is not properly utilized and is discarded or burned in open air, which results in a waste of resources and a rise in environmental concerns.

Rice husk is a high-ash biomass fuel with an ash mass fraction of 13% to 29%. The wasted rice husk ash also causes environmental pollution. It has been reported that the main component of rice husk ash is SiO_2_, which is significantly higher than other ashes, reaching up to 97.3% [3]. One should note that rice husk ash can be the cheapest source of nanostructured silica [12]. Unlike the naturally existing mineral-type crystalline SiO_2_, the rice husk-derived SiO_2_ exists in amorphous form. The amorphous SiO_2_ has shown promise in a wide range of applications, such as electronic, electrical and high-performance composite materials, due to the high specific surface area, high surface activity and purity. Rice husk ash also contains a small amount of alkali metal oxides (5–14%) besides amorphous nano-SiO_2_, which can be removed by the appropriate chemical treatment, and a silica content of >99 wt. % can be achieved [13,14,15]. Recently, several studies have focused on rice husk nano-SiO_2_ (RHNS), such as: geopolymers [16], soluble silicates [17] and amorphous SiO_2_ nanowires [18].

Poly (3-hydroxybutyrate–*co*–3-hydroxyvalerate) (PHBV) is a high concentration of microorganisms stored in cells under unbalanced particle growth conditions, such as with a lack of trace elements including nitrogen, phosphorus and magnesium. The molecular polymer can be prepared by biological fermentation or chemical synthesis from waste vegetables, fruits and plant residues. PHBV offers complete biodegradability, high biocompatibility, hydrophobicity, piezoelectricity, optical activity and high crystallinity. In addition, the excellent mechanical properties of PHBV are similar to polypropylene (PP) and are considered to be an ideal thermoplastic bio-polyester material [19]. However, PHBV has several disadvantages, such as high cost, brittle nature and easy decomposition, which limit the potential applications of PHBV [20]. Therefore, the mechanical properties and processability of PHBV should be enhanced by cost-effective processes to utilize its potential. To date, most of the studies have been focused on PHBV modification by adding inorganic or organic reinforcement materials, such as wheat straw fiber [21], carbon nanotubes [22], porous bioactive wollastonite [23] and functionalized graphene nano sheets (FGS) [24]. One should note that SiO_2_ is an excellent filler and commonly used in the fabrication of inorganic composites. Previously, RHNS has been applied to polymer reinforcements, such as high-density polyethylene (HDPE) [25], polyvinyl chloride (PVC) [26] and epoxy resin [27], to reduce the friction coefficient and improve wear resistance [28]. However, the modification of PHBV by RHNS has not been reported yet.

Herein, we have synthesized RHNS by a dry method and pre-treated with hydrochloric acid (HCl). Then, the RHNS was directly burned at 550 °C to evaporate and decompose the organic components of the rice husk and remove moisture, which resulted in nano-SiO_2_ particles. The proposed method is simple, controllable and cost-effective. In this study, the composites were fabricated by using the RHNS and PHBV and the influence of RHNS content on the mechanical properties, water absorption and thermal properties of composites was systematically investigated.

## 2. Experimental

### 2.1. Materials

PHBV (Hongyuan Plastic, Shenzhen, China), melt index (MI) 40.126 g/min, density 1.25 g/cm^3^, rice husk powder (Anhui Agricultural Products Direct Store, Hefei, China), homemade, particle size 80 mesh were used as raw materials. The RHNS content of 0, 1.5, 3, 4.5 and 6 wt. % were used to investigate the influence of RHNS content on composite performance.

### 2.2. RHNS Synthesis

To remove dirt and impurities, the rice husk was soaked in tap water for 24 h and repeatedly washed for three or four cycles. After natural drying, the rice husk was dried at 105 °C by using a blast drying oven for 24 h.

The appropriate amount of rice husk powder was placed in 5 wt. % HCl solution and heated in a water bath at a temperature of 90 °C under continuous stirring. Then, the treated rice husk powder was filtered with gauze and washed to remove acid with deionized (DI) water. These steps removed most of the metals, such as calcium, iron, potassium and other impurity ions, from the rice husk powder, which ensured the high purity of SiO_2_. Subsequently, the rice husk was dried at 105 °C for 24 h. The dried rice husk powder was placed in a crucible and heated in a low-temperature electric resistance furnace to be fully burnt in air. Then, the burnt powder was placed in a tube furnace and heated at 550 °C for 2 h to obtain an off-white rice RHNS. It has been reported that the optimum combustion temperature for the rice husk is 550 °C. One should note that the organic components cannot be completely removed at low temperature and affect the SiO_2_ color. Similarly, the product is sintered at high temperatures, resulting in the reduced specific surface area of SiO_2_ [29].

### 2.3. Fabrication of RHNS/PHBV Composites

**Figure 1:**
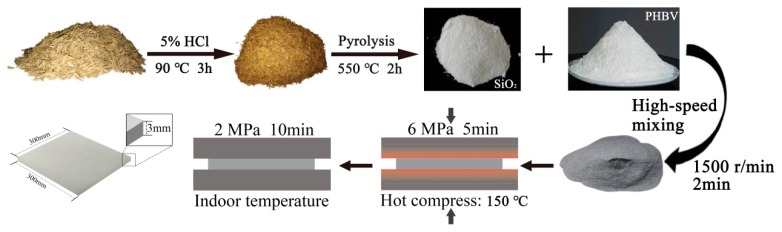
Schematic illustration of RHNS/PHBV composites fabrication.

The proper amounts of RHNS and PHBV were weighed and mixed in a high-speed mixer for 2 min at a speed of 1500 r/min. The mixture material was taken out and placed in a flat vulcanizer (800Y, Boou Hardware Products Co., Ltd., Jinhua, China), where it was pre-heated at 150 °C for 10 min and hot pressed at a pressure of 6 MPa for 5 min. Subsequently, it was placed in a cold press under the pressure if 2 MPa at room temperature for 10 min. Finally, the board was placed in a constant temperature and humidity chamber for treatment to obtain the RHNS/PHBV composites. The schematic illustration of RHNS/PHBV composites synthesis is presented in Figure 1.

### 2.4. Material Characteristics

The surface morphologies of rice husks, RHNS and RHNS/PHBV composites was observed by field emission scanning electron microscopy (FESEM, Hitachi Co. Ltd., S-4800, Tokyo, Japan) at an accelerating voltage of 2.0 kV. A layer of gold foil was deposited on the surface before SEM observations to improve the electronic conductivity and obtain high-quality images.

The crystal structure of RHNS was characterized by X-ray diffraction analysis (XRD, XRD-3, Beijing Purkinje, Beijing, China), equipped with Cu Kα radiations. The room temperature XRD patterns were recorded in the 2θ range of 5° to 60°, accelerator voltage was 36 kV and the tube current was 20 mA.

The Fourier transform infrared spectroscopy (FTIR, Bruker Model Tensor II, Bruker, Billerica, MA, USA) was carried out by using KBr pellets in the range of 400 to 4000 cm^−1^. The final scan was obtained by integrating 32 scans, measured at the resolution of 4 cm^−1^.

The tensile and flexural properties of RHNS/PHBV composites were measured using GB/T 1449-2005 and GB/T 1040-42. The mechanical tests were carried out at room temperature using a mechanical tester (Shimadzu AG-X Plus, Shimadzu Corporation, Kyoto, Japan). The reported results are an average of five independently tested specimens.

The GB/T 1462-2005 standard was used to assess the water absorption properties. The samples for the water absorption test had a size of 50 mm × 50 mm × 3 mm, and the reported result is an average of five independently tested specimens. The following equation was used to measure the weight gain:

(1) $$W=\frac{m_{1}-m_{0}}{m_{0}}\times 100\%$$

where: *W* represents the water absorption rate percentage, %; *m*_0_ corresponds to the initial weight of the sample, g; *m*_1_ refers to the weight of the wet sample, g. 

A three-dimensional sample of 20 mm × 20 mm × 3 mm was sectioned for the water swelling test. The sample was immersed in water for 24 h, and the dimensions were measured with a micrometer to the nearest 0.001 mm. The linear expansion ratio in each direction was calculated and the result was accurate to 0.0001%.

The thermal stability of prepared composites was assessed by a thermogravimetric analyzer (TGA 209F3, Netzsch, Bavaria, Germany). A 5–10 mg sample was placed in a clean Al_2_O_3_ crucible and heated within a temperature range of 30 to 700 °C at the heating rate of 10 °C/min. The TGA experimental was carried out under the constant flow of nitrogen.

The differential scanning calorimeter (DSC) was carried out by using Differential Scanning Calorimeter (DSC 200, Netzsch, Bavaria, Germany) within a temperature range of 20 to 200 °C. A 5–10 mg sample was used under the constant flow of nitrogen gas. The heating and cooling rates were 10 °C/min. The crystallinity of the composite is calculated according to the given formula [30]:

(2) $$Xc\left(\%\right)=\frac{\Delta H_{f}}{W*\Delta H_{f}(\mathrm{PHBV})}\times 100\%$$

where: *X*c refers to the crystallinity, %; ∆*H_f_* represents the melting enthalpy of composite material, J/g; ∆*H_f_ (*PHBV*)* corresponds to the 100% crystalline PHBV melting heat enthalpy, 146 J/g [31]; *W* denotes the composite mass fraction of PHBV in the material, %.

## 3. Results and Discussion

### 3.1. RHNS Characterization

**Figure 2:**
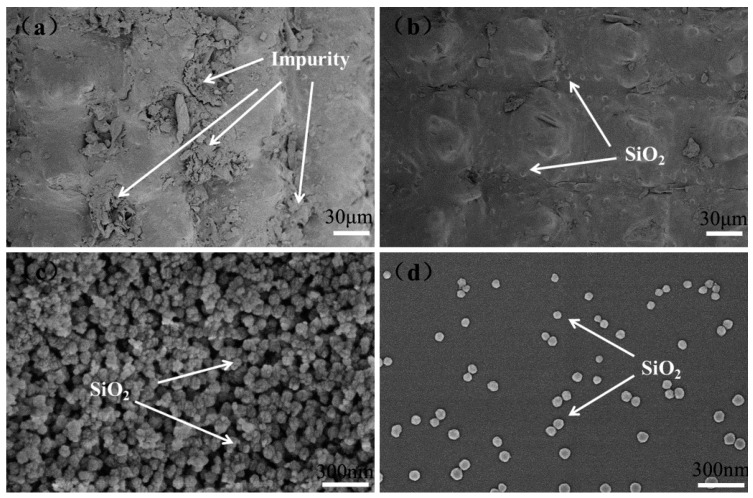
Surface morphology of rice husk and RHNS: (**a**) original rice husk; (**b**) rice husk treated with 5% HCl; (**c**) aggregated RHNS and (**d**) dispersed RHNS.

Figure 2 shows the surface morphology of the rice husk before and after pre-treatment with 5 wt. % HCl and the RHNS prepared by high temperature calcination. Figure 2a,b clearly demonstrates that water soaking and 5 wt. % HCl pre-treatment resulted in a smooth, bright and clear outer surface of the rice husk. This implies that impurities had been removed after water immersion and 5 wt. % HCl pre-treatment [32], which is important for the subsequent firing and attaining of high-purity RHNS.

Figure 2c,d presents the surface morphology of aggregated and dispersed RHNS particles. Figure 2c exhibits the spherical particles of the RHNS with a uniform particle size after high-temperature calcination. From the SEM images, the particle size of the RHNS can be estimated as being within the range of 40 to 80 nm, which is consistent with previous reports [33,34]. Moreover, the nano-SiO_2_ particles did not exhibit a horn shape and straight edges, which implies that SiO_2_ exists in the amorphous state. Hence, the amorphous RHNS can be effectively produced using the currently adopted experimental procedure. However, the fired RHNS showed a remarkably aggregated structure. A large amount of nano-SiO_2_ particles had aggregated and formed huge spherical clusters, while only a few particles remained dispersed. The aggregation was caused by the high surface energy of the nano-scale RHNS particles, which makes them highly active, unstable and aggregated.

**Figure 3:**
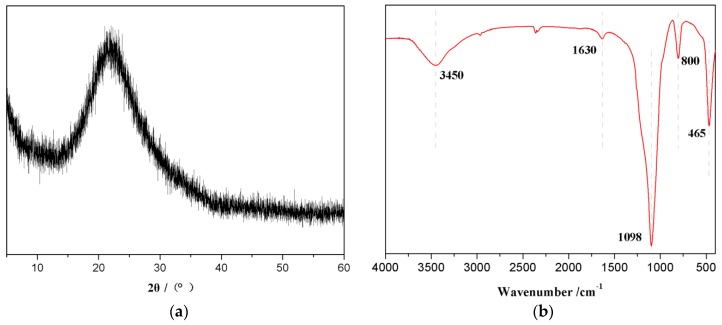
XRD pattern and the FTIR spectrum of pyrolysis obtained RHNS: (**a**) XRD pattern and (**b**) FTIR spectrum.

To determine the crystal structure of RHNS, XRD analysis was carried out and the results are shown in Figure 3a. The XRD pattern of RHNS did not show distinct diffraction peaks. Only a broad dome-shaped scattering curve appears, which is centered at 2θ = 22°. One should note that the XRD pattern is consistent with standard amorphous silica [35] and confirmed the amorphous nature of RHNS particles.

Figure 3b presents the FTIR spectra of RHNS and the major peaks appeared at 3450, 1630, 1098, 800 and 465 cm^−1^. The broad peak at 3450 cm^−1^ corresponds to the antisymmetric stretching vibrations of structural Si–OH and water–OH [36]. The peak near 1630 cm^−1^ represents the bending vibrations of H–O–H [37]. The peak near 1098 cm^−1^ denotes the antisymmetric stretching vibrations of Si–O–Si [38] and the peaks 800 and 465 cm^−1^ refer to the symmetric stretching vibrations of the Si–O bond [37,39]. The FTIR spectrum is also consistent with previously published studies [40] and confirmed the amorphous structure of RHNS.

### 3.2. RHNS/PHBV Composite Characterization

#### 3.2.1. Mechanical Properties

**Figure 4:**
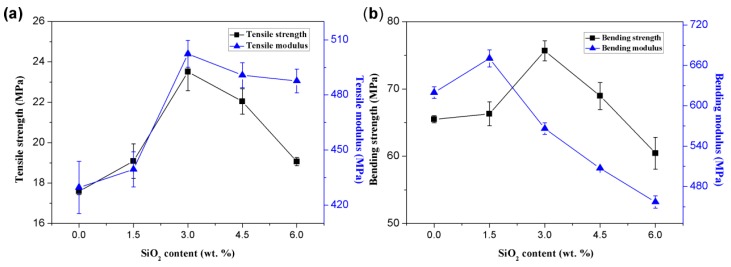
The mechanical properties of RHNS/PHBV composites: (**a**) tensile strength and tensile modulus and (**b**) bending strength and flexural modulus.

Figure 4 shows the change in mechanical properties of RHNS/PHBV composites with respect to SiO_2_ content. Figure 4a demonstrates that the tensile strength and tensile modulus of RHNS/PHBV composites increased with RHNS content up to a threshold limit, followed by a gradual decrease. The tensile strength and the tensile modulus of PHBV were 17.595 and 429.611 MPa, respectively. When RHNS content was 3 wt. %, the tensile strength and tensile modulus of the composites exhibited the maximum values of 23.515 and 502.394 MPa, respectively, which corresponds to a respective increase of 33.65% and 16.94% in comparison to pure PHBV. Once RHNS content exceeded the threshold value of 3 wt. %, the tensile strength and tensile modulus of RHNS/PHBV composites gradually decreased and exhibited the values of 19.062 and 487.633 MPa, respectively, at a SiO_2_ content of 6 wt. %. However, these values were still 8.34% and 13.51% higher than those for pure PHBV. The results demonstrate that the addition of RHNS enhanced the tensile properties of PHBV. One should also note that the lower content of RHNS showed a prominent effect due to the excellent dispersion of RHNS nanoparticles in PHBV matrix. The nano-scale SiO_2_, with its high specific surface area, enhanced the polymer crystallization [41,42] and resulted in strong interaction forces between molecular chains. However, when the content of RHNS was above a threshold limit, particle aggregation occurred, resulting in poor dispersibility, which caused the increased surface irregularities and void ratios. Moreover, the aggregation reduced the original surface effect of RHNS and resulted in a weak bonding force between nano-SiO_2_ and the matrix. In addition, the weak interfacial forces increased the interface defects and led to an inferior interfacial strength.

Figure 4b presents the influence of RHNS content on the flexural properties of the RHNS/PHBV composites. Figure 4b shows that the flexural strength and flexural modulus of the RHNS/PHBV composites increased up to a threshold limit, followed by a decrease when increasing of amount of RHNS. The flexural strength and flexural modulus of pure PHBV are 65.49 and 619.676 MPa, respectively. The maximum flexural strength of RHNS/PHBV composites was 75.669 MPa at 3 wt. % RHNS, which was 15.54% higher than pure PHBV. When the content of RHNS was 1.5%, the flexural modulus attained the highest value of 670.523 MPa, which was 8.21% higher than pure PHBV. It has been found that the addition of an appropriate amount of RHNS is required to attain desirable bending properties and enhance the rigidity of the composite materials. This may be related to the rigid nature of inorganic RHNS particles. When an appropriate amount of RHNS is added to the PHBV matrix, it entangles with the PHBV matrix and interacts with each other, thereby improving the rigidity and bending properties of composites [43]. However, an excessive amount of RHNS reduces the cross-linkage density and toughness of the composite, which negatively influences the bending strength and flexural modulus.

Furthermore, the deformation resistance of the composite materials is improved due to the excellent reinforcement effect of RHNS particles [37]. One should note that the destruction of composites requires higher energy, which corresponds to the enhanced comprehensive mechanical properties. However, once the content of RHNS exceeds a threshold value, the agglomeration and poor dispersion of nano-SiO_2_ particles in the matrix results in reduced mechanical performance.

#### 3.2.2. Water Absorption Performance

Table 1 shows the water uptake of RHNS/PHBV composites after 24 h of water immersion treatment. The water absorption of the composites has shown a relatively direct relationship with RHNS content, and a maximum value of 0.35% was attained for the composite with 6 wt. % RHNS (RHNS/PHBV-6). One should note that the water absorption capacity of pure PHBV was only 0.26%. Also, with the addition of RHNS, the water absorption of the composites slightly increased, whereas the water resistance decreased. However, the water absorption rate of the RHNS/PHBV composites was not too high, and met the general requirements of potential applications.

**Figure 5:**
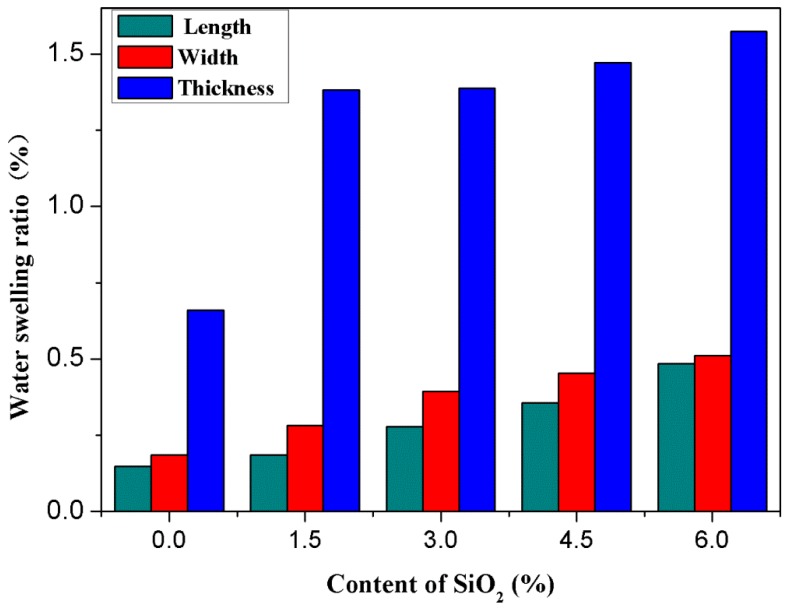
Water swelling ratio of the RHNS/PHBV composites in length, width and thickness directions.

Figure 5 shows the water swelling ratio of the RHNS/PHBV composites in the length, width and thickness directions. It can be seen from the figure that the water swelling ratio of thecomposites in three directions increased with the increasing RHNS content after water soaking for 48 h at room temperature. The length and width of the specimen increased slowly, whereas the thickness has a significant increase with respect to RHNS content. When the RHNS content increased from 0 to 6 wt. %, the water swelling ratio of length and width increased from 0.148% and 0.184% to 0.485% and 0.511%, respectively. The water swelling ratio of pure PHBV was 0.6605% in the thickness direction. When the amount of RHNS was 1.5 wt. %, the water swelling ratio in the thickness direction became 1.3819%, which is twice as high as pure PHBV. When the RHNS content continued to go up to 6 wt. %, the expansion ratio became 1.574%, which shows that water uptake had slowed down with increased RHNS content. In summary, the water swelling rate followed the given order in three directions: thickness > width > length.

The reduced water absorption performance of the composites can be ascribed to the addition of RHNS, which broke the continuity of the PHBV matrix, and increased the internal void ratio. Moreover, invisible cracks and holes appeared due to non-uniform mixing during hot pressing. One should note that the presence of holes can increase water absorption rate. In addition, the lower water absorption rate of the composites corresponds to the uniform dispersion and strong interfacial bonding of RHNS with PHBV matrix (Figure 8b–d).

#### 3.2.3. Thermal Stability

**Figure 6:**
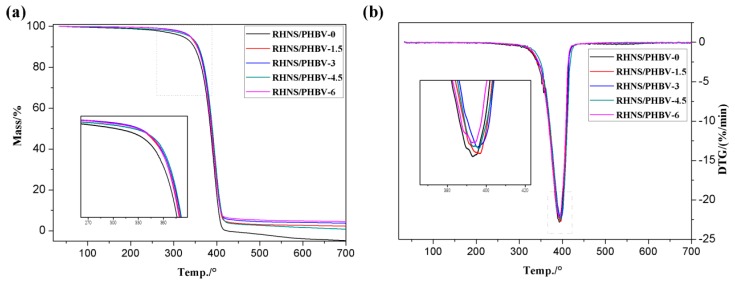
The TGA and DTG curves of RHNS /PHBV composites: (**a**) TGA and (**b**) DTG.

Figure 6 shows the temperature dependent weight loss of RHNS/PHBV composites. Table 2 summarizes the TGA data of the RHNS/PHBV composites during thermal degradation, including the initial degradation temperature (*T*_initial_) of the composite material, the temperature corresponding to the maximum degradation rate (*T*_max_), and the residual mass of the composites at 700 °C (RM at 700 °C).

The comprehensive analysis of the TGA curves revealed that the composites showed a major degradation peak, which indicates the excellent interfacial compatibility of the composites. The main degradation temperature of the composites ranged from 363 to 418 °C and the degradation rate change was in the range of 21.53% to 22.83%/min. The initial degradation temperature of pure PHBV was 363.0 °C, whereas the initial degradation temperature increased to 367.8 °C after the addition of 4.5 wt. % RHNS. *T*_max_ and RM at 700 °C also increased with increasing RHNS content, which indicates that the addition of RHNS enhanced the thermal stability of PHBV.

The improved thermal stability of the composites can be attributed to the strong interfacial bonding between the RHNS and PHBV, resulting in an increase in the energy required for degradation [44,45]. The RHNS has shown strong heat resistance and no significant degradation at 700 °C was observed. The insulating effect of RHNS helped to prevent heat transfer and delay the degradation of PHBV [46].

#### 3.2.4. Differential Scanning Calorimetry

**Figure 7:**
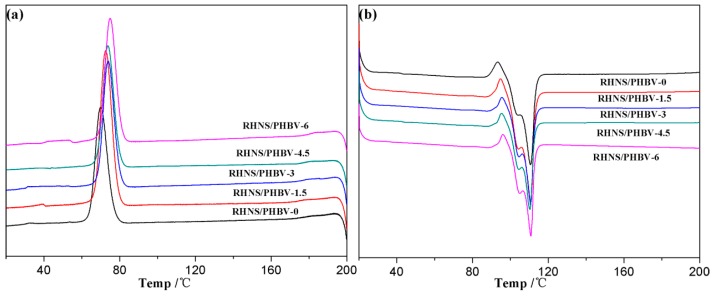
DSC curve of RHNS/PHBV composites: (**a**) cooling crystallization curve and (**b**) heating melting curve.

To eliminate the influence of the thermal history of the sample on the crystal structure, two DSC lifts and temperature tests were performed on the blend samples. Figure 7 shows the heating and cooling DSC curves of RHNS/PHBV composites with different RHNS content. The corresponding thermal analysis data are shown in Table 3. Figure 7a clearly demonstrates that the exothermic peak (*T*_c_) and the crystallization onset temperature (*T*_c,onset_) constantly moved towards the high temperature, and the highest temperature was observed at the RHNS content of 6 wt. %. The *T*_c_ and *T*_c,onset_ of pure PHBV were 70.0 and 64.6 °C, respectively. When the content of RHNS reached 6 wt. %, *T*_c_ (74.9 °C) and *T*_c,onset_ (70.0 °C) showed an increase of 4.9 and 5.4 °C, respectively. This indicates that the addition of RHNS resulted in anisotropic nucleation in the composite.

It can be seen from Figure 7b that during the heating process, the melting curves of pure PHBV and RHNS/PHBV composites showed double melting peaks, and the high-temperature melting peak represented the main region. PHBV is a semi-crystalline polymer, and the melting-recrystallization-remelting process led to the formation of a double melting peak [47]. The low-temperature melting peak (*T*_m1_) and the high-temperature melting peak (*T*_m2_) of PHBV were observed at 104.3 and 110.6 °C, respectively. The melting point of PHBV/RHNS composites ranged from 104 to 111 °C, and both melting peaks of the composite blend moved slightly towards the higher temperature. Table 3 shows that the crystallinity of the composite was higher than pure PHBV when 1.5 and 3 wt. % RHNS was added. However, when RHNS content exceeds 3 wt. %, the crystallinity of the composite becomes inferior to the pure PHBV. This can be explained by the anisotropic nucleation of RHNS, which enhanced the crystallization of PHBV. Furthermore, higher content of RHNS led to the severe agglomeration and non-uniform dispersion, which significantly influenced the anisotropic nucleation of RHNS and hindered the crystallization of PHBV. Hence, an overall reduced crystallinity of the composite was observed, resulting in a decrease in the mechanical properties.

#### 3.2.5. Ex-Situ Morphological Analysis

**Figure 8:**
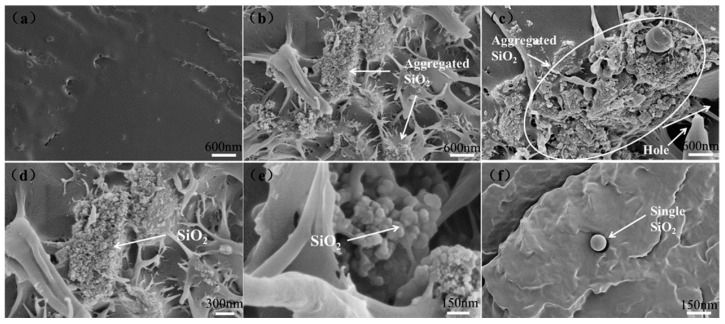
Cross-sectional SEM images of PHBV and RHNS/PHBV composites: (**a**) pure PHBV; (**b**) RHNS/PHBV-3; (**c**) RHNS/PHBV-6 and (**d–f**) RHNS morphology of composite at different magnifications.

Figure 8 presents the ex-situ SEM images of tensile cross-sections of RHNS/PHBV composites. It can be seen that the pure PHBV (Figure 8a) has a smooth and dense cross-section, which typically depicts a brittle fracture. When the RHNS content reached 3 wt. % (Figure 8b), the uniformly dispersed RHNS aggregates have been observed in the composite material. Also, the cross-section of RHNS/PHBV-3 exhibited rough and contained filamentous polymers, which results in stress dispersion. One should note that under the action of external force, the composite mainly dissipates the external force by the deformation of internal polymeric chains. When the RHNS content increased to 6 wt. % (Figure 8c), a higher concentration of large aggregates and pores was observed. Moreover, the non-uniform size distribution of RHNS/PHBV-6 composite, resulted in stress concentration of the matrix and reduced mechanical properties. The high magnification SEM images revealed that RHNS is prone to agglomeration due to the small particle size and high surface energy. Hence, a small amount of RHNS can be uniformly dispersed in the matrix and larger amounts tend to form aggregates (Figure 8b–f). A comprehensive comparison of SEM reveals that the addition of RHNS significant increases the porosity and confusion degree of the composite, which facilitates the stress dispersion and water absorption. Therefore, the SEM analysis complemented the above-mentioned results, where the RHNS/PHBV-3 composite showed optimal mechanical and water absorption properties. It is worth mentioning here that a threshold level of RHNS is required to attain the desired performance, without compromising the mechanical properties due to nanoparticle aggregation and non-uniform dispersion. In summary, RHNS/PHBV composites showed promising mechanical and water absorption properties, making them desirable candidates for a wide range of applications.

## 4. Conclusions

In summary, RHNS/PHBV composites were prepared by using rice husk-derived SiO_2_ nanoparticles and PHBV matrix. The SEM, XRD and FTIR analysis confirmed the amorphous nature of spherical nano-SiO_2_ particles, where the average diameter ranged from 40 to 80 nm. The mechanical properties, thermal stability and water absorption properties of pure PHBV and RHNS/PHBV composites have been explored and the influence of RHNS has been systematically investigated. It was found that the addition of RHNS enhanced the thermal stability of the composite and slightly increased the water absorption. The tensile strength and flexural strength of the composites initially increased up to a threshold value of RHNS content (3 wt. %), followed by a gradual decrease. SEM and FTIR were carried out to explain the enhanced mechanical properties and water absorption performance of RHNS/PHBV composites. The uniform dispersion and strong interfacial bonding of RHNS with PHBV matrix resulted in higher mechanical performance and water absorption.

Recently, the scarce nature of resources and environmental concerns require the development of novel composites, such as RHNS/PHBV, for a wide range of applications due to the biodegradable nature of PHBV and cost-effectiveness and abundance of RHNS. Hence, the present study opens up avenues for further research in various fields.

## Figures and Tables

**Table 1:** Water absorption properties of RHNS/PHBV composites with different content of RHNS.

Sample	*m*_0_/g	*m*_1_/g	*W*/%
RHNS/PHBV-0	7.99	8.011	0.26 ± 0.02
RHNS/PHBV-1.5	7.783	7.805	0.28 ± 0.01
RHNS/PHBV-3	7.659	7.681	0.28 ± 0.01
RHNS/PHBV-4.5	8.052	8.075	0.29 ± 0.01
RHNS/PHBV-6	8.255	8.284	0.35 ± 0.04

**Table 2:** Thermal analysis data of RHNS/PHBV composites derived from TGA curves.

Sample	*T*_initial_/°C	*T*_max_/°C	RM at 700 °C/%
RHNS/PHBV-0	363	393.1	0
RHNS/PHBV-1.5	365.3	396.7	2.07
RHNS/PHBV-3	366.8	395.4	3.48
RHNS/PHBV-4.5	367.8	396.2	3.79
RHNS/PHBV-6	363.8	393	4.44

**Table 3:** The DSC derived data of the RHNS/PHBV composites.

Sample	*T*_c_/°C	*T*_m1_/°C	*T*_m2_/°C	∆*H*_f_(J/g)	*X*c/%
RHNS/PHBV-0	70	104.3	110.6	65.45	44.83
RHNS/PHBV-1.5	72.6	104.4	110.6	79.42	55.23
RHNS/PHBV-3	73.9	104.5	110.7	65.98	46.59
RHNS/PHBV-4.5	73.8	104.5	110.3	60.47	43.37
RHNS/PHBV-6	74.9	105	110.8	61.28	44.65
